# Asynchronous Peer Support Chat Groups for Family Caregivers: Lessons Learned

**DOI:** 10.1093/geroni/igaf122.3716

**Published:** 2025-12-31

**Authors:** Felipe Jain, Maria Galvez, Noura Attili, Paulina Gutierrez-Ramirez

**Affiliations:** Massachusetts General Hospital / Harvard Medical School, Boston, Massachusetts, United States; Massachusetts General Hospital, Boston, Massachusetts, United States; Massachusetts Institute of Technology, Cambridge, Massachusetts, United States; Massachusetts General Hospital, Boston, Massachusetts, United States

## Abstract

Peer support groups serve as a vital resource for family caregivers of people living with dementia. Smartphone apps provide opportunities for convenient, asynchronous interaction, without the need to travel or plan for meetings. However, the value of text-based chat peer support groups for family caregivers is unclear. We aimed to determine the feasibility of a mobile App peer support chatroom embedded within a randomized, controlled trial of caregiver skills with or without mentalizing imagery therapy. Participants received the MGH CareDoc application over 24 weeks, which provided daily learning modules alongside peer support chat. For the first 8 weeks, caregivers could opt to receive weekly telehealth groups that provided psychoeducation on study topics. Caregivers were divided into chatgroups of 4-6 participants based on their telehealth group assignment and on dementia severity of the relatives, to encourage small-group participation and similarity of caregiving experiences. Feasibility and acceptability were determined based on chatroom posts and semi-structured interviews. The trial included 110 participants (mean age 68.8), divided into 29 chat groups. High variability in chatgroup activity was observed, with an average of 104 messages per chatgroup (range 1 to 904). Individual users posted an average of 29 messages (range 1 to 299). Although some participants found the chatgroups to be supportive and helpful, several participants expressed that their chat group did not fully establish cohesion. Findings highlight both potential and challenges of mobile chatrooms, and caregiver feedback on ways to improve the structure of the chatgroups.

